# Dynamic Measurement of a Cancer Biomarker: Towards In Situ Application of a Fiber-Optic Ball Resonator Biosensor in CD44 Protein Detection

**DOI:** 10.3390/s24061991

**Published:** 2024-03-21

**Authors:** Zhuldyz Myrkhiyeva, Kanagat Kantoreyeva, Aliya Bekmurzayeva, Anthony W. Gomez, Zhannat Ashikbayeva, Meruyert Tilegen, Tri T. Pham, Daniele Tosi

**Affiliations:** 1 Laboratory of Biosensors and Bioinstruments, National Laboratory Astana, Nazarbayev University, 010000 Astana, Kazakhstan; zhuldyz.myrkhiyeva@nu.edu.kz (Z.M.); abekmurzayeva@nu.edu.kz (A.B.); anthony.gomez@nu.edu.kz (A.W.G.); zhashikbayeva@nu.edu.kz (Z.A.); 2 Department of Biology, School of Sciences and Humanities, Nazarbayev University, 010000 Astana, Kazakhstan; meruyert.tilegen@nu.edu.kz (M.T.); tri.pham@nu.edu.kz (T.T.P.); 3 Department of Electrical and Computer Engineering, School of Engineering and Digital Sciences, Nazarbayev University, 010000 Astana, Kazakhstan; kanagat.kantoreyeva@nu.edu.kz

**Keywords:** dynamic measurement, in vitro detection optical fiber biosensor, ball resonator, CD44, cancer biomarker

## Abstract

The accuracy and efficacy of medical treatment would be greatly improved by the continuous and real-time monitoring of protein biomarkers. Identification of cancer biomarkers in patients with solid malignant tumors is receiving increasing attention. Existing techniques for detecting cancer proteins, such as the enzyme-linked immunosorbent assay, require a lot of work, are not multiplexed, and only allow for single-time point observations. In order to get one step closer to clinical usage, a dynamic platform for biosensing the cancer biomarker CD44 using a single-mode optical fiber-based ball resonator biosensor was designed, constructed and evaluated in this work. The main novelty of the work is an in-depth study of the capability of an in-house fabricated optical fiber biosensor for in situ detection of a cancer biomarker (CD44 protein) by conducting several types of experiments. The main results of the work are as follows: (1) Calibration of the fabricated fiber-optic ball resonator sensors in both static and dynamic conditions showed similar sensitivity to the refractive index change demonstrating its usefulness as a biosensing platform for dynamic measurements; (2) The fabricated sensors were shown to be insensitive to pressure changes further confirming their utility as an in situ sensor; (3) The sensor’s packaging and placement were optimized to create a better environment for the fabricated ball resonator’s performance in blood-mimicking environment; (4) Incubating increasing protein concentrations with antibody-functionalized sensor resulted in nearly instantaneous signal change indicating a femtomolar detection limit in a dynamic range from 7.1 aM to 16.7 nM; (5) The consistency of the obtained signal change was confirmed by repeatability studies; (6) Specificity experiments conducted under dynamic conditions demonstrated that the biosensors are highly selective to the targeted protein; (7) Surface morphology studies by AFM measurements further confirm the biosensor’s exceptional sensitivity by revealing a considerable shift in height but no change in surface roughness after detection. The biosensor’s ability to analyze clinically relevant proteins in real time with high sensitivity offers an advancement in the detection and monitoring of malignant tumors, hence improving patient diagnosis and health status surveillance.

## 1. Introduction

Despite significant progress in the field of medical research, cancer continues to pose a serious global challenge [1]. The awareness of this fact has led to an increasing amount of research and literature emphasizing the crucial significance of early detection of cancer in enhancing the efficacy of therapies [2]. The importance of early detection in improving the chances of successful outcomes in cancer treatment is being more acknowledged [3]. The monitoring of biomarkers is essential for assessing patients, directing treatment decisions, and evaluating the efficacy of medications, particularly in the field of precision medicine [4,5]. Conventional diagnostic techniques such as imaging and tissue biopsy have lower sensitivity and tend to be done in later stages of the disease, resulting in inefficiencies in medical decision-making processes [6,7]. Imaging techniques lack the capacity to precisely identify molecular activities and are insufficient for identifying slight and early alterations [6]. Whereas tissue biopsy is commonly used for cancer detection, its invasive characteristics and limited capacity to capture the dynamic aspects of the disease, like spatial variability and changes over time, are notable disadvantages [8]. On the other hand, liquid biopsies provide a least-invasive method to analyze and monitor disease [9]. It involves measuring circulating biomarkers in several body fluids, including blood, urine, saliva, and sweat, offering more convenient molecular profiling and regular monitoring [6]. An important limitation to the substantial clinical use of liquid biopsies employing biomarkers is the inconsistent detection they exhibit. The limited accuracy and reliability of findings can be attributed mostly to the small amounts of blood samples used in these examinations [10].

Cluster differentiation 44 (CD44), a transmembrane glycoprotein, is essential for cell division, motility, adhesion, and signaling [11]. It is extensively present in many types of cells, with a molecular weight of 85–200 kDa [1,12]. This protein not only contributes to physiological functions but is also becoming more closely linked to the development of cancer [12]. The interaction between CD44 and its ligands plays a vital role in the development of cancer, affecting important cellular processes like adhesion, motility, growth, and survival of cells [1].

Multiple studies have emphasized the carcinogenic features of CD44, establishing it as a crucial cancer biomarker [13]. A biomarker refers to a biological indication that could point to the possibility of developing a disease or provide information about an individual’s current health condition [14]. CD44 functions as a surface biomarker that specifically enhances the proliferation of cancer stem cells, which play a crucial role in the progression of cancer [1]. CD44 plays a crucial function in activating many signaling pathways that trigger the epithelial-mesenchymal transition, a vital mechanism that allows cancer cells to invade and spread [1]. Furthermore, the proteolytic cleavage of CD44’s transmembrane region occurs on both sides of the domain [15]. The process of cleavage, referred to as shedding, leads to an elevation in soluble CD44 in the circulation [16]. Several studies have demonstrated a positive link between elevated levels of the CD44 protein and the progression of cancer. In 1994, Guo and colleagues first noticed an elevation in soluble CD44 levels in the serum of individuals with colon and stomach [17] cancer. The researchers proposed that serum CD44s may be a valuable indicator for monitoring patient advancement, as its concentrations seemed to be closely linked to the size of the tumor [18]. Several other studies have indicated that CD44 may serve as a promising marker for the progression of cancer [19,20,21,22].

Currently, there are several methods available for the detection of soluble CD44, including photoelectrochemical (PEC) technologies that include antifouling interface biosensors, fluorescence tests, and enzyme-linked immunosorbent assays (ELISA). Research by Fan et al. attempts the development of biosensors, combining aspects of photochemical and electrochemical detection methods for biomolecules [23]. ELISA is commonly used in healthcare environments to detect biomarkers, including CD44. Research conducted on individuals with oral cancer revealed that ELISA is sensitive in detecting the CD44 protein. However, the study proposes carrying out more research on a better assay using larger patient samples to enhance the accuracy [24]. Furthermore, a study on stomach cancer samples showed that ELISA might be susceptible to cross-contamination and may not possess the same level of sensitivity as a multiplex detection test [25]. Furthermore, ELISA tests, which exhibit lower sensitivity, may not be the most appropriate choice for detecting markers present in low concentrations. The reduced sensitivity of ELISA tests makes them less suitable for detecting biomarkers that are present in minimal amounts, particularly in the initial phases of the disease [26].

Optical fiber biosensors have been employed in the detection of cancer biomarkers, achieving in situ detection [27] and rapid sensing of molecular changes at ultralow concentrations. Several fiber probes have been deployed for the detection of HER-2 (human epidermal growth factor receptor 2) [28], CCL5 (chemokine ligand-5) [29], IL-8 (interleukin-8) [30], CEACAM5 (carcinoembryonic antigen-related cell adhesion molecules 5) [31], and CK-17 (cytokeratin-17) [32].

This work is focused on the study of CD44 protein detection with an optical fiber ball resonator in vitro by employing dynamic conditions to mimic blood flow in the vein. In the previous work, the sensitivity and specificity of the developed optical fiber biosensor to detect CD44 in static conditions was demonstrated with a limit of detection at the attomolar level [33,34]. The protein was also detected in an in vitro setup mimicking the blood flow [34]. The work [34] used a commercially available catheter to integrate the sensor that was then put inside the tube with a flowing serum; since this was a proof-of-concept work, specificity studies were not performed, and the packaging system was not optimized. The current work aimed to further enhance the performance of the biosensor in the blood-mimicking system with more in-depth experiments of the in vitro setup. It includes additional study of the following: optimization of placement of the sensor inside the tube/catheter; modification of the packaging of the sensor in blood-mimicking environment; and specificity tests with control proteins and assessments of pressure insensitivity. CD44, a biomarker linked to cancer, is crucial in understanding the progression and metastasis of the disease. Therefore, accurately detecting and measuring CD44 is of significant importance for clinical diagnosis of cancer. Considering this, creating platforms for detecting CD44 is prioritized, particularly using biosensors that are both sensitive and selective. The objective of this study is to develop a packaged biosensor capable of identifying the CD44 cancer biomarker in a dynamic environment, aimed towards clinical use. This is being pursued by utilizing a custom-made fiber optic ball resonator in a laboratory setting.

## 2. Materials and Methods

### 2.1. Optical Fiber Ball Resonator Fabrication and Calibration

**Figure 1:**
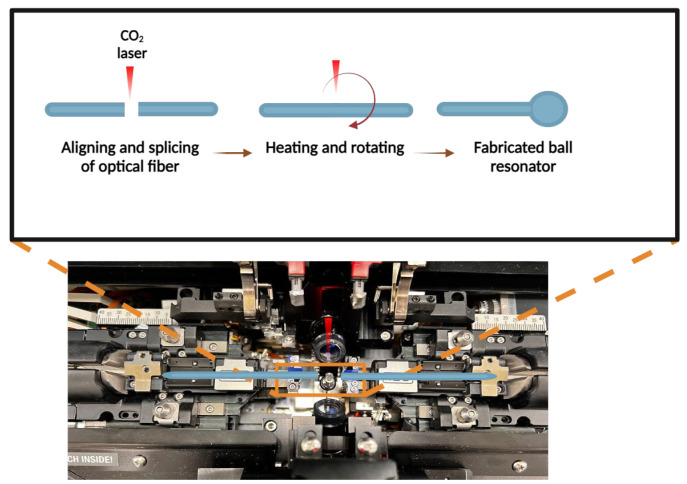
The sequential procedure of fabricating an optical fiber ball resonator using the Fujikura LZM-100. The location in the splicer where the fiber is inserted to fabricate the ball resonator is indicated in the orange box in the bottom image. The upper image shows the schematic images of the fabrication procedures, which include aligning and splicing the optical fiber before heating and rotating it with a CO_2_ laser. This process results in the formation of the ball resonator structure.

A silica single-mode fiber (SMF), SMF-28e+ (Corning, NY, USA), was utilized to construct an optical fiber ball resonator. The SMF-28 has a core size of 8.2 μm and a cladding size of 125 μm. The fabrication of an optical fiber ball resonator was completed using a CO_2_ splicing device Fujikura LZM-100 (Fujikura, Japan). Throughout the fabrication process of the ball resonator sensor, the equipment underwent calibration, and the suitable splicing mode and lens was chosen. The ball resonator’s requirements, including its diameter, were adjusted using the Fiber Processing Software. The Fujikura LZM-100 was used to create a spherical ball lens by heating and spinning the optical fiber. Precise modifications were carefully done to optimize the size and quality of the ball resonator, including adjustments to its power, rotation speed, and feeding speed. The instrument was set up, and two prepared SMF-28 were aligned and joined using a CO_2_ laser. The process involved the stripping, cleaving, and positioning of the fibers within the device. The fabrication procedure involved aligning, splicing, heating, and rotating the fibers, which resulted in the formation of a spherical resonator at the end of the fiber. The diameter of the resonator was around 500 μm. Figure 1 illustrates the fabrication process of the optical fiber ball resonator from SMF-28. The length of the fabricated sensor was approximately 12 cm. Then it was stripped and spliced using a splicing machine to attach the pigtail connector. Finally, the ball resonator was spliced and connected to the optical backscatter reflectometer.

Ball resonators of determined diameters are further assessed for their reflective properties and sensitivity to the refractive index (RI) change. The assessments were performed with a LUNA OBR 4600 optical backscatter reflectometer, which is coupled with a computer for data analysis. The OBR detects the light reflected by the ball resonator device over the wavelength range 1535–1610 nm, using an optical frequency-domain reflectometer setup; the settings of the OBR are resolution bandwidth 0.25 GHz, scan range 85 nm, spectral data filtered using a digital low-pass filter with 0.08 cut-off. When choosing ball resonators, priority was given to those that have a high sensitivity to RI. Calibration involves the use of sucrose solutions that exhibit established RI values. The calibration was conducted in both static and dynamic conditions, using the same sucrose concentrations but different volumes for the dynamic analyses. The dynamic calibration of fabricated optical fiber ball resonators was done using sucrose solutions with varying concentrations; 30 mL amount of a 10% sucrose solution was used as the first calibration point for the first RI point. Following the initial measurement of RI, further measurements were taken by gradually adding 2, 4, 6, and 8 mL of a 40% sucrose solution to the 30 mL of a 10% sucrose solution. RI values increased from 1.34761 to 1.35696 in response to changes in the concentration of the sucrose solution. The sensor’s sensitivity was evaluated by calibrating it under both static and dynamic conditions using identical concentrations. By conducting calibration on the identical sensor in both static and dynamic situations, a comparative assessment of sensitivity is attained.

With this fabrication method, each ball resonator is characterized by a quasi-random optical spectrum; in order to compare different sensors, we perform an analysis similar to [34], determining the intensity change of the most significant spectral feature (peak or valley in the spectrum).

**Figure 2:**
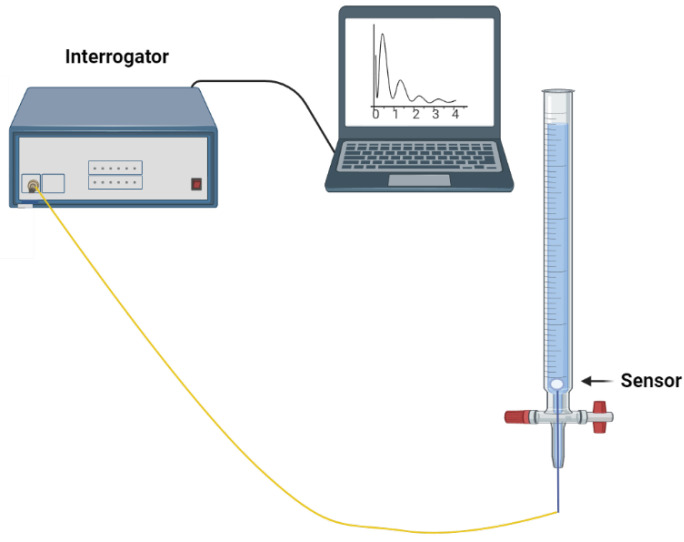
Pressure characterization setup for an optical fiber ball resonator. A 499 μm resonator is placed into the tip of a burette and then systematically filled with DI water at different levels. The pressure data recorded at water column heights ranging from 16 cm to 66 cm demonstrates that changes in pressure do not alter the detected signals.

In order to verify that the signals received were not caused by changes in pressure, a study was done to characterize the pressure on an optical fiber ball resonator. The resonator, with 499 μm size was inserted into a burette through its tip and subsequently sealed with clay to ensure no leakage occurs. Deionized (DI) water was thereafter poured into the burette in a systematic manner from the top. Throughout this procedure, pressure measurements were recorded at several water column elevations, specifically at 16, 26, 36, 46, 56, and 66 cm. The method and arrangement, which involves the placement of the burette and the resonator, are illustrated in Figure 2.

### 2.2. Optical Fiber Ball Resonator Surface Functionalization

After calibrating the sensors, ball resonators were chosen for the functionalization step based on their reflectivity and sensitivity to RI change. Initially, the sensor was pre-treated using Piranha solution for 15 min. Piranha solution constituted of sulfuric acid and hydrogen peroxide, composing a 1:4 ratio, followed by meticulously washing with DI water. After eliminating the organic residues and increasing the surface hydroxyl groups via Piranha solution, the resonators were treated with 1% solution of (3-aminopropyl)trimethoxysilane (APTMS) for a duration of 20 min for silanization. Following the APTMS treatment, the resonators were washed using methanol and then exposed to a one-hour heat treatment in an oven maintained at a temperature of 110 °C. After undergoing heat treatment, optical fiber ball resonators were washed with DI water and subsequently immersed in a 25% glutaraldehyde solution, which serves as cross-linker for antibody immobilization in the next steps, for a duration of 1 h.

Following the initial functionalization steps, the surface of the ball resonators was prepared for the attachment of CD44 monoclonal antibodies (Sigma-Aldrich, Darmstad. Germany, No SAB4700179) using a targeted concentration of 4 μg/mL. The antibody immobilization step lasted 1 h of continuous shaking to ensure uniform antibody immobilization across the ball resonator’s surface. After attaching the CD44 antibodies, the resonators were placed in a solution containing 10% poly(ethylene glycol) methyl ether amine (mPEG amine). The purpose of this 30-min immersing was to inhibit any non-specific binding sites. Afterwards, the ball resonators were washed with PBS and then stored in PBS in the fridge at a temperature of 4–8 °C, ready to be used for protein measurement.

### 2.3. Dynamic Protein Measurement

**Figure 3:**
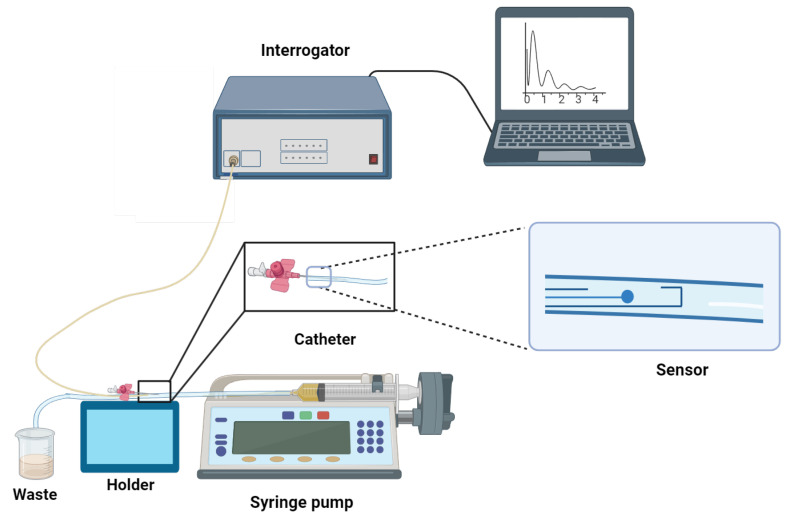
An illustration of the setup for the dynamic measurement of the CD44 protein. The setup includes a Legato 100 KD Scientific syringe pump, which operates at a flow rate of 20 mL/min to mimic venous circulation, and a 20-gauge polyurethane cannula that protects the optical fiber ball resonator, coupled with a LUNA OBR 4600 device for accurate detection.

An in vitro detection system was developed to mimic the blood circulation in veins for measuring CD44 protein levels. The system used a syringe pump, Legato 100 KD Scientific, that had the capability to provide adjustable flow rates. The pump’s flow rate was set at a constant value of 20 mL/min for imitating blood flow in veins. The CD44 protein, with concentrations ranging from 7.1 aM to 16.7 nM, was passed through a thin tube with a diameter of 1 mL at the specified flow rate. Before performing protein measurements, the internal part of the experimental tubing was blocked by treating it with a 1% bovine serum albumin (BSA) solution. The measurement of the CD44 protein was performed using a diluted calf serum at the ratio of 1:10. CD44 protein with concentrations of 16.7 nM,12.9 pM, 9.3 fM, 7.1 aM and a total volume of around 60 mL each were prepared for dynamic measurement. The specificity tests were done with two control proteins: thrombin and gamma globulin. The measurement setup consisted of a 20-gauge (G) cannula made of polyurethane (PUR); it was sealed on the tip to protect the inserted optical fiber ball resonator, and one segment of the top part of the cannula was removed. The length of this catheter was 32 mm, and its interior diameter was around 1.1 mm. The biosensor was firmly placed within the tube using a tricot bevel needle to create a hole. The sensor was connected to the LUNA OBR 4600 device (Luna Inc., Roanoke, VA, USA). A schematic illustration of this in vitro system is shown in Figure 3.

With respect to prior works, the packaging hereby designed accomplishes a three-fold objective: (1) Allow the fiber positioning in line with respect to the vessel along which the detection takes place; (2) prevent the measurement artefact due to the presence of the outer walls mimicking a blood vessel; (3) prevent the measurement artefact due to the packaging itself acting over the ball resonator. The configurations used in prior studies were designed for static tests, whereas this catheter is optimal for dynamic measurement since it prevents the artefacts due to the relative motions of the ball resonator with respect to the blood vessel and to the catheter itself. Since the ball resonator is a sensor with very low reflectance, the placement of sensors in this way allows for obtaining a clean measurement even during the motions due to the pumping system.

### 2.4. The Surface Morphology Study

The analysis of sensor morphology at every stage of functionalization was carried out with a Bruker Nanowizard 4XP Atomic Force Microscope (AFM), which was combined with an inverted ZEISS Axio Observer 7 microscope. For the purpose of the study, three optical fiber ball resonators were taken after each step of the functionalization process and placed into PBS solution before being examined. The AFM investigation utilized a Bruker AFM Respa20 probe, which is characterized by a resonant frequency range of 13 to 27 kHz and a force constant ranging from 0.45 to 1.8 N/m. The measurements were conducted in a liquid environment (PBS) utilizing a contact mode QI approach. Three distinct scans were conducted for each sensor, covering a 1 μm^2^ region. The RMS Roughness of these regions was later computed.

## 3. Results and Discussion

### 3.1. Fabrication of Optical Fiber Ball Resonators and Their Calibration in Dynamic Conditions

The primary objective of this study was to examine the possibility of developing an optical fiber biosensor with the ability to detect the CD44 protein in dynamic conditions. For the detection of CD44 cancer biomarker in a dynamic system, a ball resonator was fabricated from single-mode telecommunication fiber by using a CO_2_ laser and interrogated with the OBR device to confirm sensitivity to the changes in the RI. Interrogating the fabricated optical fiber ball resonators allows for the assessment of the sensitivity and quality of the response generated by the fabricated sensors to changes in the RI of the surrounding medium. Optical fiber ball resonators of varying diameters are shown in Table S1. The study relied on data analysis, employing the MATLAB program (MathWorks, Natick, MA, USA). The two-dimensional profilometry of the fabricated optical fiber ball resonator is shown in Figure S1.

**Figure 4:**
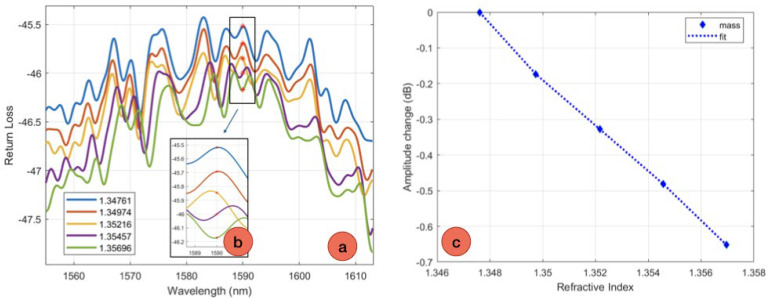
Example of a calibration of a ball resonator for RI detection. (**a**) Spectrum of the ball resonator probe, for various RI values. (**b**) Inset displaying the spectrum in proximity of the detected spectral feature. (**c**) Change of intensity as a function of the refractive index.

The efficacy of the sensors was assessed by examining their reflection spectra and the corresponding amplitude variations. Sensors with high sensitivity to RI change were selected for the biofunctionalization process to further protein detection steps. Even decrease in amplitude was observed across sensors when the RI values increase. This indicates that there is a consistent linear relationship for each sensor since their coefficient of determination (R^2^) is more than 0.99. To detect the CD44 protein these optical fiber ball resonators were subjected to further functionalization, which resulted in their transformation into specialized biosensors that were particularly designed to detect CD44. Dynamic sucrose calibration was carried out, and the results showed that there was no distinct difference between the two sets of values. Table 1 provides a comparison of the calibration data for sucrose concentration using two different sensor sizes, including 527 μm and 514 μm, in both static and dynamic conditions; Figure 4 shows the example of calibration of a ball resonator, while Table S2 shows the splice parameters used on the Fujikura LZM-100 for the device fabrication. The table provides a quantitative measure of the sensitivity of each sensor, indicating that both sensors maintain an equivalent degree of sensitivity under both dynamic and static situations. The sensitivity values are displayed in conjunction with the coefficient of determination (R^2^), which indicates a high degree of prediction accuracy for calibrating sucrose concentration. The sensor with a diameter of 527 μm demonstrates a sensitivity of −83 dB/RIU. It shows a correlation to the predicted findings, as evidenced by a R^2^ value of 0.99 under static settings and a slightly lower but still good R^2^ of 0.95 under dynamic conditions. Similarly, the 514 μm sensor demonstrates a sensitivity of −86 dB/RIU in static conditions with an R^2^ value of 0.99. In dynamic conditions, the sensor exhibits a sensitivity of −85 dB/RIU, with an R^2^ value of 0.95. The resolution of refractive index detection is estimated as 2.4 × 10^−4^ RIU. The numbers highlight the strength and consistency of the sensors’ performance in various testing circumstances. The decrease in the coefficient of determination could be a result of the 20 mL/minute flow rate inside the tubing during the dynamic measurement that was mimicking blood flow.

The fabrication method proposed hereby allows the rapid manufacturing of ball resonators based on single-mode telecom fibers, each characterized by a quasi-random spectrum and a low bill-of-material cost (considering the fiber cost is lower than $0.1/m). By using the recipe stored on the CO_2_ laser splicer, the diameter of ball resonators varies from 485 μm to 514 μm (average: 501.8 μm; standard deviation: 15.3 μm), with RI sensitivity varying from 83 dB/RIU to 128 dB/RIU (average: 95.9 dB/RIU; standard deviation: 13.5 dB/RIU). In addition, Figure S2 shows the response of the sensor to a step concentration change.

**Figure 5:**
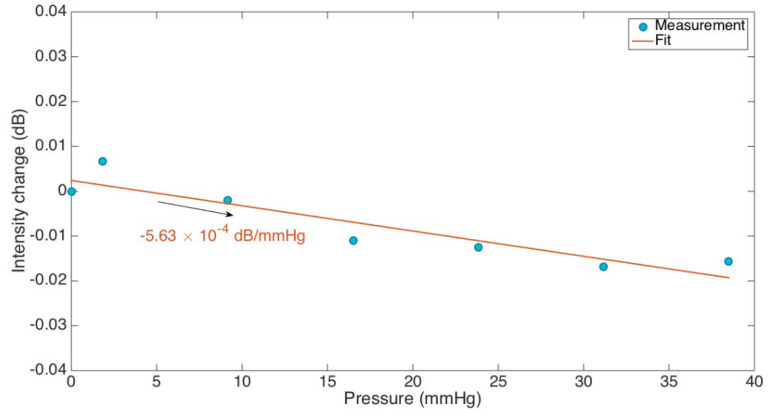
Analysis of pressure effects on the 499 μm optical fiber ball resonator.

To demonstrate the optical fiber ball resonator sensor’s resistance to pressure changes, its response was tested at various water depths, using a sensor with a diameter of 499 μm. The sensor was exposed to different pressures in a controlled experiment that simulated various water depths. These pressures were measured as follows: 9.9 mmHg, 11.73 mmHg, 19.07 mmHg, 26.4 mmHg, 33.74 mmHg, 41.07 mmHg, and 48.4 mmHg. The objective was to monitor the sensor’s sensitivity to changes in pressure, which is a crucial determinant for circumstances that simulate blood circulation. The data points on the graph in Figure 5 correspond to the sensor’s change in intensity at different pressures, while the fitted line indicates a low sensitivity of −5.63 × 10^−4^ dB/mmHg. This suggests the sensor’s performance is not affected by differences in pressure. This result is important since it confirms that the sensor will continue to maintain its accuracy when used in medical devices or similar applications, in spite of the fluctuating pressure circumstances commonly seen in vascular systems. The results indicated that the sensor did not respond to changes in pressure. This evaluation was particularly important for its application in environments simulating blood flow, where varying blood pressures are a factor, to ensure that the sensor remained unaffected by pressure changes.

### 3.2. Surface Morphology of the Functionalized Optical Fiber Ball Resonators by AFM

The Atomic Force Microscope (AFM), a type of Scanning Probe Microscope (SPM), operates based on the variation in interaction force between a sample and a cantilever. This cantilever, equipped with a pointed probe at its end, bends upon interacting with the sample’s surface [35]. Such a mechanism enables the AFM to examine the surface’s morphology and various properties. The resolution of the images produced by AFM can reach atomic levels, depending on the probe tip’s radius, allowing for the analysis of sample characteristics like elasticity, deformation, adhesion, and energy dissipation [36]. In this study, the surface morphology of the ball resonator was analyzed using a JPK Nanowizard 4XP AFM (Bruker Instruments, Germany) in conjunction with an inverted ZEISS Axio Observer 7 microscope (ZEISS Instruments, Germany) to assess variations in the surface morphologies at all functionalization stages. A RESPA20 probe (Bruker, Germany) with a nominal spring constant K = 0.9 N/m, resonant frequency f = 20 kHz, and radius r = 8 nm was subjected to the scanning parameters of 0.5nN setpoint, z-speed of 40 m/s, and scan rate of 1 Hz. Prior to obtaining several 1 μm × 1 μm images with a resolution of 5nm/pixel in the X-Y plane, a scanning region of 10 μm × 10 μm was utilized to probe the ball’s broad view and evaluate the surface homogeneity. All measurement were performed using QI Biomolecules in liquid mode in PBS at room temperature. Gwyddion software was used to visualize the images. For each functionalization step, a total of three independent samples were obtained, each with at least 15 different 1 μm × 1 μm patches for statistical analysis. GraphPad Prism 10 was used to perform statistical tests to properly measure the dispersion in surface height and root-mean-square (RMS) roughness, as well as the statistical significance of the results between distinct phases.

**Figure 6:**
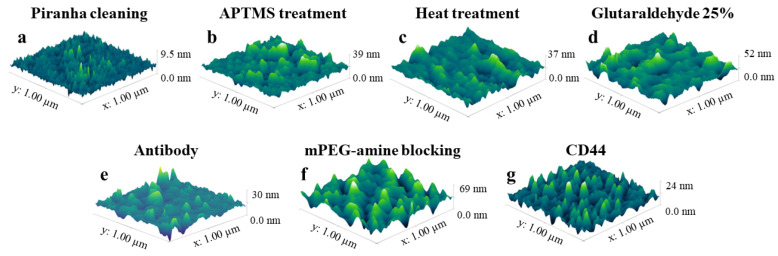
Representative images of the biosensors’ surface morphologies obtained from AFM measurements at various stages of functionalization. Images of different stages of functionalization in a 1 μm × 1 μm section of an optical fiber ball resonator’s surface are displayed: (**a**) Piranha pre-treatment, (**b**) silanization with APTMS, (**c**) heat treatment, (**d**) cross-linking with GA, (**e**) immobilization of antibodies, (**f**) blocking with mPEG-amine, and (**g**) CD44 protein detected by a ball resonator.

Figure 6 show the representative surface morphologies (3D images) of the sensor surfaces at various functionalization phases. These images reveal that each treatment step alters the surface morphology. For instance, the process of silanization with APTMS increases surface roughness, while subsequent treatment with GA smoothens it, as also noted by Shaimi et al. [37]. GA, commonly used in functionalization, links the amino groups on the surface (from APTMS) to the ligand’s amines, such as those in proteins like antibodies [22]. After the CD44 antibodies are immobilized on the sensor, mPEG-amine is used to block nonspecific sites, attaching via its amine group, and presenting a non-reactive methoxy group on the surface.

**Figure 7:**
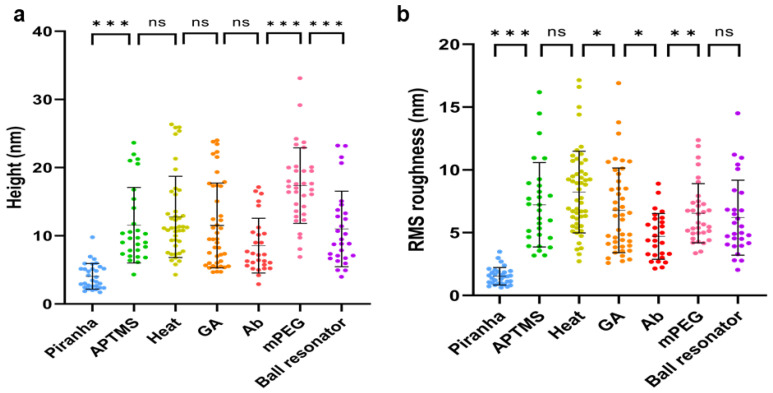
Quantitative analysis of the height and surface roughness of various functionalization phases for optical fiber ball resonator biosensors. Assessing the variations in height (**a**) and RMS-roughness (**b**) at every stage of functionalization. * *p* ≤ 0.05, ** *p* ≤ 0.01, *** *p* ≤ 0.001, ns, *p* > 0.05.

Figure 7 presents the statistical findings of height and root mean square (RMS) roughness assessments on biosensor surfaces for all functionalization phases: Piranha pretreatment, APTMS silanization, heat treatment following silanization, GA incubation, antibody immobilization, mPEG-amine blocking, and CD44 protein detected ball resonator. For all phases, the mean and standard error of the mean (SEM) values of the height and root-mean-square (RMS) roughness are calculated and summarized in Table S3. As can be observed, the measured roughness value of 1.53 ± 0.12 nm indicates that the surface of the bare sensor treated with Piranha in Figure 6a is rather smooth. The surface roughness of the sensor was raised to 7.22 ± 0.62 nm by APTMS treatment, as shown in Figure 6b. Figure 6c shows that heat treatment has little effect on surface roughness, 8.45 ± 0.50 nm. Following subsequent glutaraldehyde (GA) treatment, the surface roughness decreased marginally to 6.77 ± 0.51 nm, as seen in Figure 6d. Upon immobilization of CD44 antibodies, the roughness did not increase further; instead, it dropped to 4.70 ± 0.35 nm (Figure 6e). The surface being conjugated with CD44 antibodies fills in the valleys made by the GA treatment, reducing the height differential between the surface’s peaks and troughs, causing the surface roughness to decline as a result. Blocking the surface with mPEG-amine further raised the height and enhanced the surface roughness to 7.82 ± 1.03 nm as demonstrated in Figure 6f. When CD44 proteins were introduced to the ball resonator for detection, the height significantly dropped but the surface roughness changed only slightly to 6.19 ± 0.56, which was not significantly different from the surface treated with mPEG-amine (see Figure 6g). The biosensor is so sensitive that it can detect when only a few CD44 proteins have adhered to the sensor surface, which is insufficient to enhance the surface roughness of the entire scan region. It is worth mentioning that AFM measurements of dried samples of the optical biosensor used to detect the cancer biomarker CCL5 yielded comparable results [29]. Here, all surface morphology assessments were conducted in PBS under physiological conditions, which replicates the actual behavior of the protein and surface in their natural environments during detection. Our findings thus demonstrated that it is possible to image the surface morphology of a biosensor in an aqueous solution while maintaining the sensitivity and accuracy of AFM measurements.

### 3.3. CD44 and Control Proteins Measurements

In the previous work, the detection of CD44 in static conditions was demonstrated with a determined limit of detection at the attomolar level, which is around six times more sensitive compared to commercially available ELISA kits [34]. The work also included a preliminary study of CD44 protein detection in a dynamic setup. In this study, this dynamic setup was further enhanced to detect CD44 protein in conditions simulating blood flow. The efficiency of optical fiber ball resonators in capturing CD44 was evaluated in dynamic conditions, as CD44 is commonly found in circulating blood. Therefore, for precise diagnosis and prognosis of the disease, the analysis of a substantial volume of blood is essential. This was facilitated by a system in which a catheter, integrated with an optical fiber ball resonator, was inserted into tubing carrying diluted calf serum. The serum flowed at a rate of 20 mL/min, mimicking blood flow. The structure of the commercially available catheter was refined to meet the purpose of this study (Figure 3). Namely, removing the catheter’s upper region around 5–7 mm near the catheter’s end while keeping the bottom part intact with sealed tip to enhance its performance in two ways: (1) allowing the biomarker to be in contact with the functionalized sensor; (2) keeping the sensor from breaking. This optimization allowed it to lower the artifact and hence to detect the protein concentration in dynamic setup rather than the wall of the tube (i.e., blood vessel). During the CD44 detection assay, an increase in signal correlating with the protein concentration was observed, confirming the resonators’ capability to detect CD44 at extremely low concentrations, building upon findings from previous research [33]. Building a fiber optic biosensor that would allow direct detection of the protein in the blood has advantages over the existing methods of protein detection such as ELISA or electrochemical biosensor. A biosensor generates a signal as the analytes interact with the sensor and do not require labeled reagents or multi steps for measuring the analyte. Using a ball resonator as a transducer in biosensor would allow its use directly in the bloodstream since the optical fiber is biocompatible and does not exhibit electromagnetic interference.

**Figure 8:**
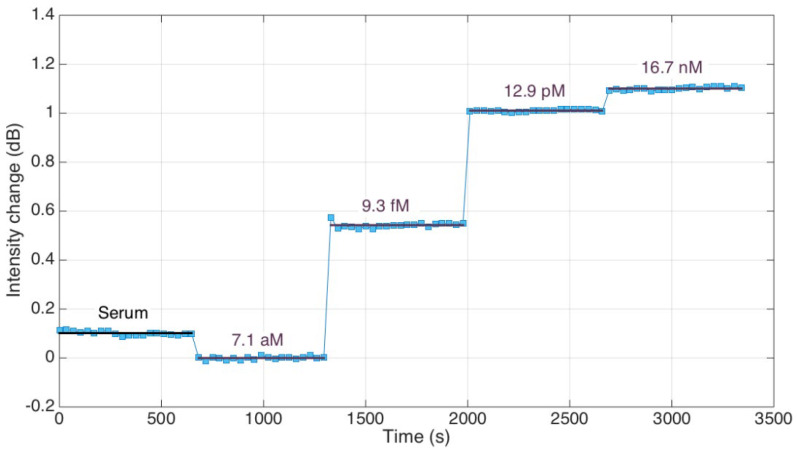
An optical fiber ball resonator biosensor’s efficacy in detecting CD44 was analyzed. The sensorgram shows the change in signal intensity over time, showing how a functionalized optical fiber ball resonator reacts to increasing amounts of CD44 in serum, ranging from 7.1 aM to 16.7 nM in diluted calf serum; results from the 497 μm diameter sensor are highlighted.

The graph in Figure 8 is a sensorgram that measures the detection of CD44 protein in a dynamic flow system as time progresses. The x-axis represents the passage of time, measured in seconds, ranging from 0 to around 3500 s. The y-axis quantifies the variation in intensity in decibels (dB), spanning from −0.2 to 1.4 dB. From the left side, the graph displays a consistent baseline at the beginning, which remains steady when serum is introduced, showing no noticeable alteration in intensity. The baseline period is essential as it sets the sensor’s initial state prior to the introduction of the CD44 protein. Over time, there are clear and apparent increases in intensity that occur in distinct steps at regular intervals. Each step represents the sensor’s response to a particular concentration of CD44 protein. The concentrations are indicated on the graph at the specific points of change, beginning at 7.1 aM and increasing steadily to 9.3 fM, 12.9 pM, and finally 16.7 nM. The existence of these well-defined levels demonstrates the sensor’s high sensitivity and its capacity to differentiate between various levels of CD44 concentration. The low fluctuation in the data for each concentration tested suggests a high level of stability and precision in the sensor measurements. Accurate measurement is crucial for ensuring that the sensor functions consistently in a controlled laboratory environment.

**Figure 9:**
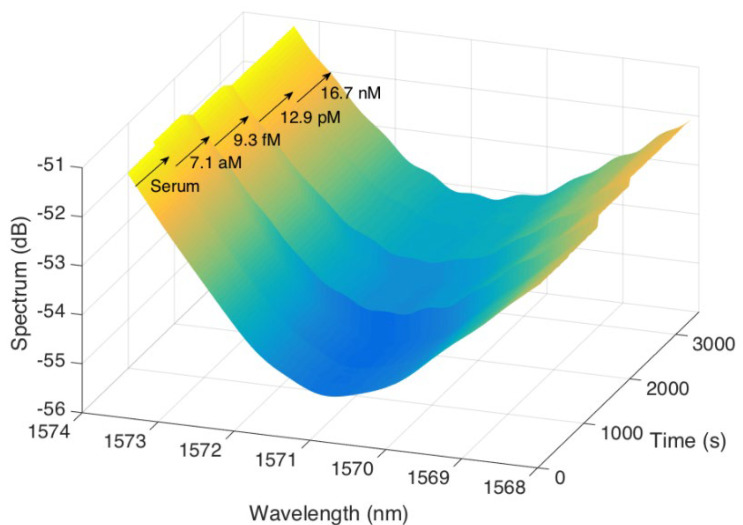
This 3D sensorgram shows the real-time detection of CD44 using a biosensor with a 497 μm optical fiber ball resonator. The sensorgram plots the spectral intensity against wavelength and time. The color gradient change from blue to yellow visually demonstrates the biosensor’s response to increasing concentrations of CD44 in serum, which range from 7.1 aM to 16.7 nM. Each peak correlates to a specific CD44 concentration, demonstrating the sensor’s ability to measure.

Figure 9 shows the real-time detection of CD44 using a 497 μm optical fiber ball resonator biosensor in a 3D format that aims to demonstrate the sensor’s performance. The x-axis represents time in seconds, ranging from 0 to 3000 s, to record the duration of the detecting procedure. The y-axis represents the wavelength in nm, spanning from 1568 nm to 1574 nm, showing the biosensor’s spectral detection range. The z-axis indicates the spectral strength in decibels (dB), ranging from −51 dB to −56 dB. The graph demonstrates the progressive response of the biosensor to different concentrations of CD44. This result not only demonstrates the sensor’s ability to differentiate between different concentrations but also illustrates the measurement of CD44 over a period of time. The sensorgram is crucial for evaluating the practicality of the biosensor in a research or clinical environment, providing valuable information on its sensitivity and specificity. The graph’s peaks and valleys demonstrate the optical fiber ball resonator’s interaction with CD44 at different concentrations and summarize the biosensor’s ability to detect within the measured range.

**Figure 10:**
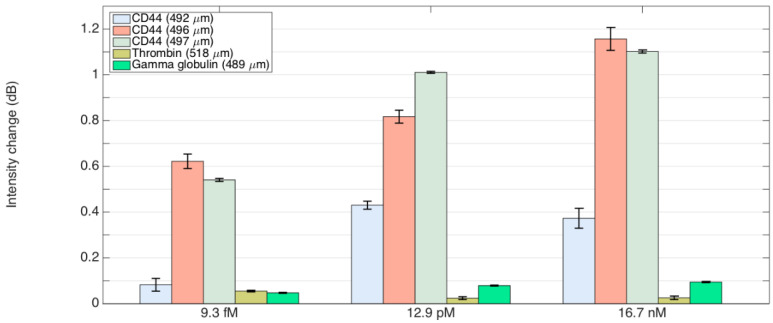
Evaluation of optical fiber ball resonator biosensors’ specificity for CD44 in comparison to thrombin and gamma-globulin. The bar graph illustrates how biosensors with diameters varying from 492 to 497 μm responded differently to CD44, while showing minimal responses to gamma-globulin (489 μm) and thrombin (518 μm) at concentrations ranging from 9.3 fM to 16.7 nM. This indicates the biosensor’s specific affinity for CD44. Error bars represent the measurements standard deviation, emphasizing the consistency and reliability of the sensor’s specificity.

Figure 10 shows a bar chart of the responses of optical fiber ball resonator biosensors to varying concentrations of CD44 for thrombin and gamma globulin. The data clearly reveal that biosensors with diameters of 496 μm and 497 μm exhibit a significant amplification in signal intensity when exposed to CD44 with concentrations ranging from 9.3 fM to 16.7 nM. The biosensor with a slightly smaller diameter of 492 μm is only sensitive to protein concentrations of 12.9 pM or higher. The response at 9.3fM of CD44 concentration is around 0.1 dB, which is indistinguishable from that of the control proteins thrombin and gamma globulin. In contrast, responses to thrombin (518 μm) and gamma globulin (489 μm) for all concentrations tested are around 0.1 dB or lower, indicating the low binding affinity of the control proteins to the developed biosensor. This graph shows the size-dependent sensitivity and specificity of biosensors to CD44 when compared to other proteins, illustrating its potential and limitations for sensitive biological applications.

**Figure 11:**
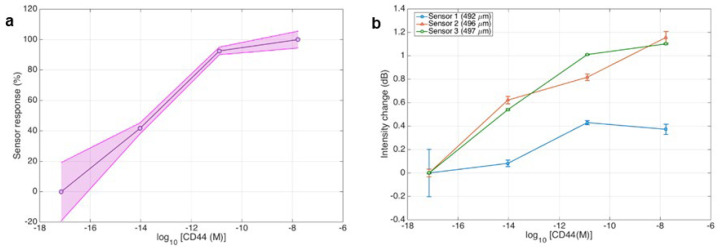
Results for CD44 detection from three biological replicates. (**a**) The graph shows the sensor’s response percentages in a logarithmic range of CD44 concentrations, demonstrating the sensors’ consistent performance in several trials. This is visible from the overlapping data points and the shaded confidence interval. (**b**) The graph compares intensity change (dB) for three sensors with varying diameters (492 μm, 496 μm, and 497 μm) to the logarithmic concentration of CD44. It indicates that the sensors operate consistently when CD44 levels increase. Error bars are used to indicate the standard deviation, which highlights the accuracy of the sensors while taking several measurements.

Figure 11a demonstrates the repeatability of sensor performance in detecting CD44 at various levels of concentration. The sensor’s output, represented as a percentage, is graphed against the logarithmic scale of CD44 molar concentrations. Three separate traces correlate to three independent sensors, each shown by a line within the darkened region, which indicates the range of responses or margin of error. The proximity of these lines indicates a level of consistency in the sensor data, as seen by the shaded region which represents the uniformity of the response throughout the sensors. Figure 11b shows how three distinct sensors (492 μm, 496 μm, and 497 μm) respond to varying CD44 concentrations. The results confirmed that the 492 μm sensor has inferior sensitivity compared to the other two with greater diameters. Thus, the findings indicate that larger diameter ball resonator biosensors should be employed when detecting protein concentrations in the femtomolar range or lower.

## 4. Conclusions

In this study, the detection of the CD44 cancer biomarker under dynamic conditions was reported using a single-mode optical fiber ball resonator biosensor. The goal of this work was to develop and assess a biosensing platform that is both economical and simple to produce, with the ability to detect a specific cancer biomarker of interest, such as the CD44 protein in real-time. The method for fabricating the sensor is both cost-efficient and time-effective, taking only five minutes, thanks to the use of cost-effective SMF-28 optical fiber, which is widely used in telecommunications. The primary objective of the study is to establish a link between laboratory settings and clinical procedures by replicating physiological variables, including blood circulation. The constructed optical fiber ball resonator biosensors were shown to be insensitive to changes in pressure and highly specific to CD44 protein. While mimicking intravenous biomarker detection it was important to examine pressure sensitivity as changes in pressure can significantly affect the detection process potentially masking biomarker signals. The fabricated sensors didn’t show sensitivity to pressure changes. Additionally, changes in protein concentration allow for almost instantaneous detection, emphasizing its potential for real-time tracking applications. Placement of the sensor inside the tube/catheter of the sensor inside the packaging was studied in more detail in this study and optimal position was chosen which didn’t create artefact during protein measurement. The packaging of the biosensor was also enhanced to improve its applicability in two ways: (1) allowed the sensor to be in contact with the fluid (and therefore biomarker); (2) kept the sensor from breaking. The proposed packaging, validated in a flowing system, did not introduce any measurement artefact while maintaining the capability for resolving concentrations of CD44. The results indicate a femtomolar detection limit when using a ball resonator that has at least 496 μm in diameter. AFM measurements were also performed to track the surface morphology of the biosensor during its various functionalization stages. The AFM data further support the sensor’s extreme sensitivity by showing a considerable shift in height but no change in surface roughness after detection. The specificity of the biosensor has been further confirmed by control studies with thrombin and gamma-globulin since no significant changes in the signal were seen in the presence of these proteins. Given that the performance of sensors might be compromised under flow settings, it is an important accomplishment to demonstrate in dynamic conditions a good reaction to the target molecule or protein with minimal signal to other proteins present in the bloodstream [26]. In situ detection of soluble CD44 biomarker in blood-mimicking conditions could be considered as a significant step forward towards building clinical devices able to detect biomarkers in blood flow.

## Figures and Tables

**Table 1:** A comparison of two different sensor sizes’ sensitivity readings for a ball resonator under static and dynamic conditions (527 μm and 514 μm).

Sensor Diameter	Sensitivity in Static Condition	Sensitivity in Dynamic Condition
527 μm	−83 dB/RIU R^2^ 0.99	−83 dB/RIU R^2^ 0.95
514 μm	−86 dB/RIU R^2^ 0.99	−85 dB/RIU R^2^ 0.95

## Data Availability

Data will be available upon request.

## Supplementary Materials

The following supporting information can be downloaded at: https://www.mdpi.com/article/10.3390/s24061991/s1, Figure S1: The geometrical 2D profile of a ball resonator 492–485 μm fabricated by Fujikura LZM-100; Figure S2: Evaluation of the kinetic response of the ball resonator biosensor to a step change in concentration. The 10–90% response time is estimated as 61.4 s; Table S1: Optical fiber ball resonator data; Table S2: Splice parameters for ball resonators; recipe for Fujikura LZM-100 splicer; Table S3: Mean height and root-mean-square (RMS) roughness of the sensor’s surface determined from the obtained AFM height images (n ≥ 40).
