# Supplementary material for: Dynamic Measurement of a Cancer Biomarker: Towards In Situ Application of a Fiber-Optic Ball Resonator Biosensor in CD44 Protein Detection
Source: Sensors (Basel). 2024 Mar 21;24(6):1991. doi: 10.3390/s24061991 (PMC10974573; doi:10.3390/s24061991)
Supplement: Supplementary file 1 [file sensors-24-01991-s001.zip › sensors-2833993-supplementary.pdf]

sensors

MDPI

Article

Dynamic Measurement of a Cancer Biomarker: Towards In Situ Application of a Fiber-Optic Ball Resonator Biosensor in CD44 Protein Detection

Zhuldyz Myrkhiyeva [1,2], Kanagat Kantoreyeva [3], Aliya Bekmurzayeva [1], Anthony W. Gomez [1], Zhannat Ashikbayeva [1,2], Meruyert Tilegen [2], Tri T. Pham [2] and Daniele Tosi [1,3,*]

1 Laboratory of Biosensors and Bioinstruments, National Laboratory Astana, Nazarbayev University, 010000 Astana, Kazakhstan; zhuldyz.myrkhiyeva@nu.edu.kz (Z.M.); abekmurzayeva@nu.edu.kz (A.B.); anthony.gomez@nu.edu.kz (A.W.G.); zhashikbayeva@nu.edu.kz (Z.A.)
2 Department of Biology, School of Sciences and Humanities, Nazarbayev University, 010000 Astana, Kazakhstan; meruyert.tilegen@nu.edu.kz (M.T.); tri.pham@nu.edu.kz (T.T.P.)
3 Department of Electrical and Computer Engineering, School of Engineering and Digital Sciences, Nazarbayev University, 010000 Astana, Kazakhstan; kanagat.kantoreyeva@nu.edu.kz
* Correspondence: daniele.tosi@nu.edu.kz

Citation: Myrkhiyeva, Z.; Kantoreyeva, K.; Bekmurzayeva, A.; Gomez, A.W.; Ashikbayeva, Z.; Tilegen, M.; Pham, T.T.; Tosi, D. Dynamic Measurement of a Cancer Biomarker: Towards In Situ Application of a Fiber-Optic Ball Resonator Biosensor in CD44 Protein Detection. *Sensors* **2024**, *24*, 1991. https://doi.org/10.3390/s24061991

Academic Editors: Hugo Aguas, Flavio Esposito and Benjamin L. Miller

Received: 4 January 2024
Revised: 15 March 2024
Accepted: 19 March 2024
Published: 21 March 2024

Copyright: © 2024 by the authors. Licensee MDPI, Basel, Switzerland. This article is an open access article distributed under the terms and conditions of the Creative Commons Attribution (CC BY) license (https://creativecommons.org/licenses/by/4.0/).

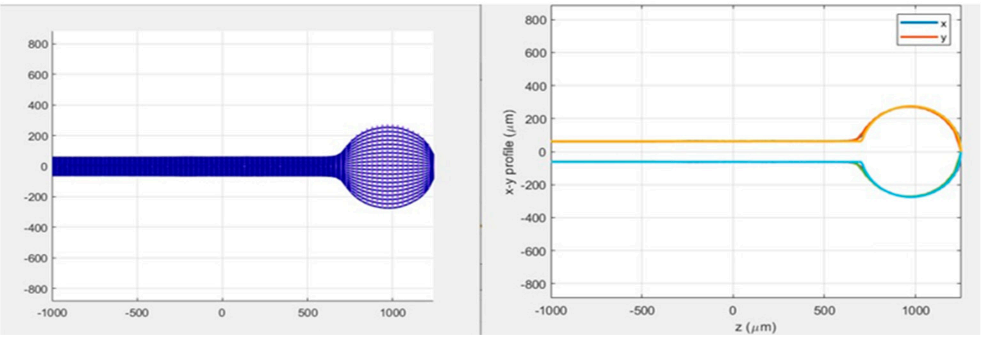

Figure S1. The geometrical 2D profile of a ball resonator 492-485 μm fabricated by Fujikura LZM-100.

Table S1. Optical fiber ball resonator data.

	Sensor	Reflection dB/mm	Sensitivity (dB/RIU)	R^2	Purpose
**1**	489 µm	air: 35<br>water: 47	**-96**	**1.00**	Gamma globulin detection
**2**	491 µm	air: 35<br>water: 48	**-90**	**0.96**	CD44 detection
**3**	492 µm	air: 37<br>water: 47	**-98**	**0.95**	CD44 detection
**4**	496 µm	air: 35<br>water: 45	**-83**	**0.99**	CD44 detection
**5**	497 µm	air: 35<br>water: 50	**-98**	**0.95**	CD44 detection
**6**	499 µm	air: 36<br>water: 48	**-85**	**0.97**	CD44 detection
**7**	521 µm	air: 37<br>water: 49	**-128.37**	**0.98**	CD44 detection
**8**	533 µm	air: 36<br>water: 46	**-105**	**0.97**	CD44 detection
**9**	485 µm	air: 37<br>water: 50	**-83**	**0.98**	Thrombin detection
**10**	518 µm	air: 44<br>water: 54	**-105**	**0.95**	Thrombin detection
**11**	499 µm	air: 36<br>water: 48	**-84**	**0.97**	Pressure measurement

Table S2. Splice parameters for ball resonators; recipe for Fujikura LZM-100 splicer.

Parameter	Settings
Pre-heat	1
Absolute power	342
Relative power	150
Feeding speed	0.2 mm/s
Rotator speed	150 deg/s
Diameter adjustment	10 μm

Table S3. Mean height and root-mean-square (RMS) roughness of the sensor's surface determined from the obtained AFM height images (n ≥ 40).

Treatment	Piranha	APTMS	Heat	GA	AB	mPEG	Ball resonator
Height (nm)	4.05±0.34	11.56±4.1.05	12.77±0.91	11.51±0.95	8.55±0.76	17.36±0.95	11.0±1.05
RMS roughness (nm)	1.53±0.12	7.22±0.62	8.45±0.50	6.77±0.51	4.70±0.35	7.82±1.03	6.19±0.56

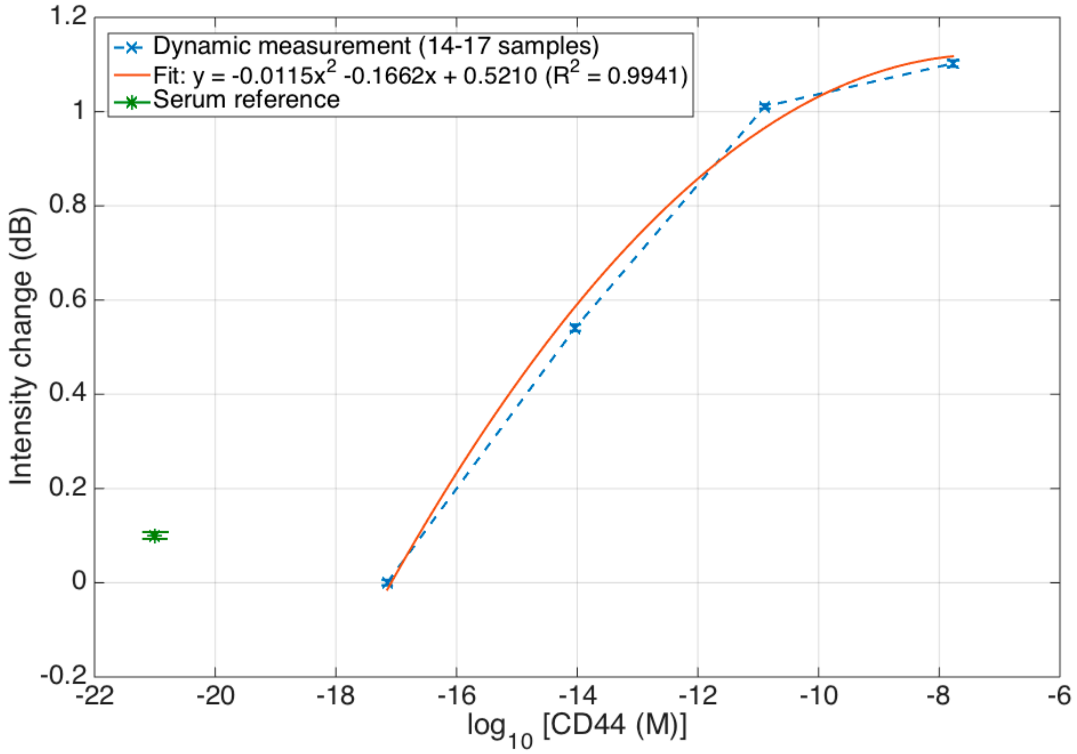

Figure S2. Output of the CD44 biosensor, reporting the intensity change for each concentration, and the blank sample. The sensitivity is obtained by log-quadratic fit. The estimated limit of detection is 2.93 fM, using the analytical method LoD = $f^{-1}(x_{blank} + 3\sigma_{max})$, where: f(x) is the log-quadratic fit, x_{blank} is the blank sample, σ_{max} is the maximum of the standard deviation for each concentration.

Disclaimer/Publisher's Note: The statements, opinions and data contained in all publications are solely those of the individual author(s) and contributor(s) and not of MDPI and/or the editor(s). MDPI and/or the editor(s) disclaim responsibility for any injury to people or property resulting from any ideas, methods, instructions or products referred to in the content.
